# Correction to ‘Locomotor compromises maintain group cohesion in baboon troops on the move’

**DOI:** 10.1098/rspb.2022.0276

**Published:** 2022-03-09

**Authors:** Roi Harel, J. Carter Loftus, Margaret C. Crofoot

*Proc. R. Soc. B* **288**, 20210839. (Published online 28 July 2021) (doi:10.1098/rspb.2021.0839)

In the paper Roi Harel, J. Carter Loftus and Margaret C. Crofoot 2021. Locomotor compromises maintain group cohesion in baboon troops on the move. *Proc. R. Soc. B* 288, 20210839. http://doi.org/10.1098/rspb.2021.0839

The required change is to change the first institution for all of the authors from Department of Ecology, Evolution and Behavior, The Life Sciences Institute, The Hebrew University of Jerusalem, Givat Ram, Jerusalem 9190401, Israel to Department for the Ecology of Animal Societies, Max Planck Institute of Animal Behavior, Bücklestraβe 5, Konstanz 78467, Germany.

